# Barriers and facilitators to promoting evidence uptake in Chinese medicine: a qualitative study in Hong Kong

**DOI:** 10.1186/s12906-021-03372-5

**Published:** 2021-07-15

**Authors:** Charlene Hoi Lam Wong, Jeffrey Van Ho Tse, Per Nilsen, Leonard Ho, Irene Xin Yin Wu, Vincent Chi Ho Chung

**Affiliations:** 1 Jockey Club School of Public Health and Primary Care, Prince of Wales Hospital, Shatin, Hong Kong; 2 Department of Health, Medicine and Caring Sciences, Linköping University, Building 511-001, Entrance 76, plan 13, Campus US, 58183 Linköping, Sweden; 3 School of Chinese Medicine, Chung Chi College, The Chinese University of Hong Kong, Shatin, Hong Kong; 4 Department of Epidemiology and Health Statistics, Xiangya School of Public Health, Central South University, Rm 527 5/F, 238 Shangmayuanling Alley, Kaifu District, Changsha, China

**Keywords:** Consolidated framework for implementation research (CFIR), Evidence-based healthcare (EBHC), Traditional, complementary and integrative medicine (TCIM), Qualitative study

## Abstract

**Background:**

In response to the World Health Organization’s recommendation, policy makers have been adopting evidence-based healthcare approach to promote the development of traditional, complementary and integrative medicine (TCIM) into Hong Kong’s health system. Disseminating synopses of clinical evidence from systematic reviews or randomized trials is regarded as a potentially effective strategy to promote evidence uptake. The study aimed to identify barriers and facilitators to implementing this strategy among Hong Kong Chinese medicine practitioners (CMPs).

**Methods:**

Twenty-five CMPs aged under 45 years and trained in Hong Kong after reunification with China in 1997 were interviewed individually. Four clinical evidence synopses of randomized trials and systematic reviews on Chinese medicine interventions were presented, and CMPs were asked to comment on their applicability in routine practice. The Consolidated Framework for Implementation Research (CFIR) was applied to guide interview and analysis.

**Results:**

The barriers included: i) CMPs’ perceived difficulties in applying complex evidence in decision-making and ii) inadequate training and limited consultation time. The facilitators were i) availability of publicly accessible and user-friendly synopses, ii) formation of community of evidence-based practice among CMPs with input from key opinion leaders, iii) opportunity for interprofessional collaborations with conventional healthcare providers, and iv) patients’ demand for evidence-based clinical advice. Besides, i) CMPs’ knowledge and beliefs in evidence-based healthcare approach, ii) presentations of evidence-based information in the synopses, and iii) clinical decision making as influenced by quality of evidence reported acted as both barriers and facilitators.

**Conclusions:**

This CFIR-based qualitative study investigated how the World Health Organization recommendation of promoting evidence use in routine practice was perceived by CMPs trained in Hong Kong after reunification with China in 1997. Key barriers and facilitators to applying evidence were identified. Such results will inform tailoring of implementation strategies for promoting evidence uptake, in the context of a well-developed health system dominated by conventional medicine.

**Supplementary Information:**

The online version contains supplementary material available at 10.1186/s12906-021-03372-5.

## Background

The World Health Organization (WHO) Traditional Medicine Strategy 2014–2023 advocates the integration of traditional, complementary and integrative medicine (TCIM) into national health systems among member states through the promotion of its evidence-based use [1]. Chinese Medicine (CM), as a major form of TCIM in Hong Kong, has received Beijing’s support for development since reunification with China in 1997 [2]. With its colonial history, Hong Kong’s health system is dominated by conventional medicine. Echoing the WHO’s strategic recommendation, the Government of Hong Kong Special Administrative Region (SAR) has been adopting the evidence-based healthcare (EBHC) approach to promote the integration of CM [2]. EBHC refers to a systematic approach to clinical problem solving which allows the integration of the best available research evidence with clinical expertise and patient values [3, 4]. In Hong Kong, CM practice is under full statutory regulation, and the private sector is the major CM service provider [5]. To promote education and research in CM, the government has established 18 semi-public CM clinics which are operating on a self-financed basis with government subsidy [6].

The EBHC approach regards systematic reviews (SRs) and randomized controlled trials (RCTs) as the best study designs to generate or summarize evidence on treatment effectiveness of CM [7]. In the past decades, there has been an increasing number of CM trials published in major international journals [7]. Clinical evidence is growing and becoming increasingly available for informing decision-making among CM practitioners (CMPs). Nevertheless, there are several barriers which might inhibit CMPs’ use of clinical evidence in routine practice. A survey conducted in China indicated that CMPs had difficulty in acquiring evidence from full reports of RCTs and SRs, lack of time and knowledge in interpreting the evidence, and expressed concerns about the trustworthiness of the studies [8]. In the UK, a qualitative study showed that CMPs had limited understanding of the concept of EBHC and related technical terminologies [9]. In Hong Kong, the level of government support to CM clinical research and education remains low when compared with other healthcare disciplines [6]. A previous study suggested that tailored EBHC training for Hong Kong CMPs might facilitate implementation of EBHC in routine CM practice [10].

To address the aforementioned barriers, the use of synopses of SRs and RCTs may also be considered. Synopses are structured abstracts of pre-appraised studies that support clinical actions without the need for the clinicians to access or digest the full article [11]. They can improve patient care by providing clear and concise evidence-based clinical information at a timely manner [11]. This approach is widely adopted in conventional medicine, with established examples such as the JAMA Clinical Guidelines Synopsis platform [11]. Synopses may thus be used to promote evidence uptake in routine practice [12]. A SR on information retrieval tool reported that the likelihood of making an evidence-based decision was influenced by the ease of information retrieval effort by clinicians [13]. Synopses may facilitate this process by reducing information retrieval burden. For example, according to a prospective observational study, evidence-based synopses delivered as daily emails were found to positively influence clinicians’ inclination in adopting evidence [14].A freely accessible website presenting synopses of SRs and RCTs on CM interventions has recently been launched to explore how CMPs response to such evidence dissemination approach [15].

While the WHO Traditional Medicine Strategy 2014–2023 is advocating TCIM development in an evidence-based manner, little is known about the faciliators and barriers to clinical evidence application in TCIM practice. The Hong Kong case of CM development provides an opportunity to investigate how this strategic recommendation is implemented in a well-developed health system. In this qualitative study, we investigated Hong Kong CMPs’ perceptions of applying results from evidence-based synopses in their routine practice from an implementation science perspective. The objective was to identify key barriers and facilitators to using results from such synopses in CMPs’ routine practice. Implications from these findings may inform future development of potential implementation strategies for enhancing evidence uptake among CMPs.

## Methods

### Study design

In-depth individual interviews were conducted during two periods with 25 CMPs aged below 45 years. All participants were trained in Chinese medicine in Hong Kong after reunification with China in 1997. The first phase of interviews was carried out among 15 CMPs in April 2017. Upon completion of analysis, we noticed that further interviews should be conducted to advance our understanding on CMPs’ perceptions towards the application of results from evidence-based synopses in their routine practice more comprehensively. Hence, second phase of interviews was performed among 10 CMPs in April 2020. Results generated from additional data confirmed that saturation was reached with no new perspectives being discovered when the sample size reached 25. A qualitative approach was chosen because little is known about facilitators and barriers to using evidence in routine CM outpatient practice in Hong Kong. Interviews were considered as the most relevant method for collecting exploratory information and gaining a deeper understanding of this issue.

**Fig. 1:**
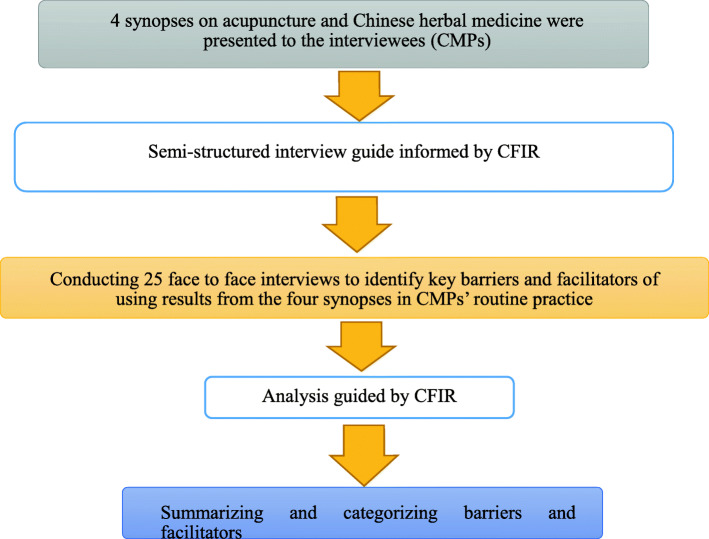
Flowchart of the study. Keys: CFIR, The Consolidated Framework for Implementation Research; CMP(s), Chinese medicine practitioners

The discussion focused on four critically appraised evidence-based synopses on CM interventions, including the most commonly applied modalities of Chinese herbal medicine and acupuncture (Additional file 1). These four synopses were sent to the participants by email and they were requested to assess their real-world applicability prior to the individual in-depth interviews. The interviews were conducted in Cantonese with the aim of identifying key barriers and facilitators to using results from the four synopses in CMPs’ routine practice. A flowchart describing steps of the study is available in Fig. 1.

### Study population, sampling and setting

A purposive approach was used to recruit a heterogeneous sample of Hong Kong CMPs, which included those with and without prior EBHC training as well as varying years of clinical experience, so as to strengthen the diversity of perspectives in this study [16]. CMPs with prior EBHC training referred to those who completed a full module of training in EBHC. We achieved a 100% response rate to invitations. We recruited CMPs from two investigators’ alumni networks (VCHC and JVHT), sampling graduates from two different Schools of Chinese Medicine in Hong Kong. Sociodemographic and professional characteristics of participants are presented in Table 1.

**Table 1:** Sociodemographic and professional characteristics of Chinese medicine practitioners (*n* = 25)

Characteristics	Values
**Demographics**	
Gender, Female, n (%)	12 (48.0)
Age range (years)	24–41
**Education and Practice**	
**Highest education level attained**	
Doctoral degree, n (%)	7 (28.0)
Master’s degree, n (%)	12 (48.0)
Bachelor’s degree, n (%)	6 (24.0)
Received prior EBHC training, n (%)	12 (48.0)
**Years of practice**	
Less than 5 years, n (%)	12 (48.0)
5–10 years, n (%)	6 (24.0)
11–15 years, n (%)	7 (28.0)
**Employment**	
**Employment status**	
Part-time, n (%)	4 (16.0)
Full-time, n (%)	21 (84.0)
**Types of work settings**	
Private CM clinics, n (%)	7 (28.0)
Semi-public CM services	
- NGO CM clinics, n (%)	3 (12.0)
- Tripartite CMCTRs, n (%)	9 (36.0)
- University CM clinics, n (%)	5 (20.0)
- Others: CM volunteer, n (%)	1 (4.0)

Keys: *EBHC* evidence-based healthcare, *CM* Chinese medicine, *NGO* non-governmental organizations, *CMCTRs* Chinese Medicine Centers for Training and Research

Note: The semi-public tripartite CMCTRs are operated by Hong Kong Hospital Authority (HA, the public tax funded health system), local universities and NGOs

Before the start of the interview, participants were informed of the purpose of the study, confidentiality of their contribution, their voluntary participation and right of withdrawal at any time. Each individual interview was conducted face-to-face by one main interviewer (VCHC or CHLW) and one research personnel (JVHT) either at the CMP’s clinic or at the University. VCHC is a Hong Kong registered CMP with more than 15 years of experience in clinical epidemiology, EBHC teaching and research. CHLW is a public health researcher with significant experiences in conducting qualitative interviews and analysis, while JVHT is a registered CMP with qualitative research experiences. Written informed consent was obtained from participants and all of them agreed to participate in the study. The study was approved by the Survey and Behavioral Research Ethics Committee, The Chinese University of Hong Kong, Hong Kong [reference no.: 101–16].

### Theoretical framework and data collection

A semi-structured interview guide (Additional file 2) was developed by VCHC and CHLW, as informed by the Consolidated Framework for Implementation Research (CFIR). CFIR is a widely used determinant framework in implementation science [17]. It is based on 20 theories, models and frameworks from various fields, including implementation science, research utilization and organizational theory [17]. The framework offers a set of constructs which can be customized for research in diverse scenarios and settings [17]. The CFIR includes 39 implementation constructs across 5 overarching domains (i.e. types of determinants of implementation outcomes): intervention characteristics, outer setting, inner setting, characteristics of individuals, and implementation process [17]. The constructs and domains are interdependent and various interactions may influence implementation of evidence in practice [18].

Each interview lasted for 50–60 min. During the interviews, VCHC or CHLW led and asked the interview questions, and JVHT took field notes. All interview content was recorded and transcribed verbatim. In case there were discrepancies or misunderstanding on content of the interviews, audio recording was used to verify interpretations, with discussion among investigators.

### Data analysis

The interview content was analyzed in relation to the five domains of CFIR in the interview language (Cantonese) iteratively as the interviews proceeded. First, two investigators (CHLW and JVHT) were responsible for coding the transcribed interviews based on CFIR using NVivo software [19]. Important sentences and words in the transcript were coded into relevant domains and constructs of the CFIR, via a deductive approach. These sentences and words were then summarized into short statements which captured key concepts and thoughts. These short statements were used to compare core concepts within the CFIR domains. To reduce researcher bias, the interpretation of codes and statements were discussed among VCHC, CHLW and JVHT, of which they have different professional backgrounds so as to reduce researcher bias. Discrepant coding was revised until consensus was reached [16]. At the end of analysis, selected representative quotes were translated from Cantonese to English. All CFIR domains, except implementation process, were applied in the analysis as the current study did not focus on actual program execution.

## Results

Analysis of the interview data yielded findings that could be mapped onto nine constructs in four CFIR domains: characteristics of individuals (one construct). intervention characteristics (three constructs), inner setting (three constructs), outer setting (two constructs). These constructs acted as barriers or/ and facilitators to using results from the four evidence-based synopses in routine practice, in accordance with the statements from Hong Kong CMPs (numbered 001–025) (Table 2).

**Table 2:** Analysis of the interviews: Consolidated Framework for Implementation Research (CFIR) domains, constructs and description of the constructs

CFIR domains and domain descriptions	CFIR constructs	Descriptions of constructs
*I) Characteristics of individuals:* It refers to the characteristics of CMPs in Hong Kong who participated in this study.	Knowledge and beliefs about the intervention [F/B]^a^	CMPs’ attitudes toward, and values placed on the use of results from synopses in routine practice.
*II) Intervention characteristics*: It refers to the characteristics of critically appraised evidence-based synopses on CM interventions which were presented to Hong Kong CMPs.	Relative advantage [F/B]^a^	CMPs’ perception of the advantage of using results from synopses, relative to their routine practice.
	Complexity [B]^a^	CMPs’ perceived difficulty of using results from synopses in routine practice.
	Design quality and packaging [F/B]^a^	CMPs’ perceived excellence in how synopses are bundled, presented, and assembled.
III) *Inner setting:* It refers to the political and cultural contexts with the local CM industry, including both private and non-private sectors.	Readiness for implementation - Available resources [B]^a^	The level of resources dedicated for using results from synopses in CMPs’ routine practice and its on-going operations. These include training, education and time.
	Readiness for implementation - Access to knowledge and information [F]^a^	CMPs’ ease of access to digestible information and knowledge about the use of results from synopses in routine practice.
	Networks and communication [F]^a^	The nature and quality of webs of social networks and the nature and quality of formal and informal communications within the local CM industry on the use of results from synopses in routine practice.
IV) *Outer setting:* It includes economic, political and social context within the Hong Kong health system.	External policy and incentives [F]^a^	External strategies and incentives for spreading synopses within the Hong Kong health system.
	Patient needs and resources [F]^a^	The extent to which patient needs influence CMPs’ use of results from synopses in routine practice, as well as resources devoted to meet those needs.

Key: Consolidated Framework for Implementation Research, CFIR; CM, Chinese medicine; CMPs, Chinese medicine practitioners

^a^The four domains of CFIR presented in this study include intervention characteristics, outer setting, inner setting and characteristics of individuals involved. The constructs of implementation determinants act as barriers [B] or/ and facilitators [F]. [B] are factors that are perceived to inhibit the use of results from synopses in routine practice based on the CMPs’ statements, while [F] are factors that are likely to promote the use of results from synopses in CMPs’ routine practice. Determinants which act as both facilitators and barriers are labelled as [F/B]

### Characteristics of individuals

#### Knowledge and beliefs about the intervention

CMPs with prior EBHC trainings were able to comprehend results reported in the synopses, and they had more positive attitudes towards application of evidence in routine practice: *“Previous EBHC training allowed me to have a better understanding on study methodology and quality of evidence, so I am more confident to interpret the results of synopses myself and decide whether to apply them in my routine clinical practice.” [021]*Other CMPs who held neutral attitudes towards synopses application would consider CM treatments described in the synopses when they face challenges in improving patient outcomes: *“I like the concept of synopses, but I still prefer using traditional syndrome differentiation for individualizing my treatment plan as I am not familiar with interpreting research evidence. Nevertheless, I would still consider these so called evidence-based treatments described in synopses if I don’t see any improvement after my first line treatment, or under circumstances where I don’t feel confident about using syndrome differentiation in designing the initial treatment plan.” [017]* Also, synopses which summarized newly generated and continually updated clinical evidence were favored by CMPs in circumstances where they need to handle uncertainty in clinical practice: *“Synopses provide the best available clinical evidence which guide me to recognize and manage diseases that I have not dealt with before in my clinical practice.” [016]* However, as evidence on the management of familiar condition may evolve, it is unclear whether CMPs would also find this important.

### Intervention characteristics

#### Relative advantage

Quality of evidence reported in each synopsis assisted CMPs in making clinical decisions. CMPs noticed that “*Synopses reporting high quality of evidence would increase my confidence in using CM treatments stated in the synopses.” [018]* Interestingly, some CMPs did not consider quality rating as an important aspect for making adoption judgment. Besides, they stated that *“In CM, individual case reports or case series were often regarded as key reference among us, as it was perceived that they have summarized thousand years of positive clinical experiences.” [001]* Other CMPs claimed that *“Synopses were fundamentally different, as they have summarized findings of existing peer-reviewed RCTs and SRs, offering much more trustworthy evidence.” [009]* Nevertheless, CMPs without relevant EBHC training seemed to ignore their differences in terms of internal validity: *“Synopses were quite similar to case series as both summarize CM clinical evidence.” [004]* This reflected a lack of understanding on the basics of clinical research methods as well as principles of EBHC.

#### Complexity

CMPs usually prescribed individualized CM treatments for patients based on syndrome differentiation. However, some CMPs indicated that *“RCTs and SRs seldom investigated effectiveness of individualized CM treatments in managing a specific disease, due to limited external and model validity.” [007]* They thought that synopses summarizing findings of studies without syndrome differentiation were not compatible with their routine practice: *“A patient’s CM diagnosis is affected by many factors, including geographic regions and climates, and therefore highly individualized treatment will be prescribed accordingly. However, biomedicine places priority on treatments standardization, and therefore influencing how CM clinical research is being done. The standardized treatments described in the synopses of existing RCTs or SRs may therefore not be directly applicable in Hong Kong context as we prefer the individualized approach.” [012]* This showed that the complexity of CM syndrome differentiation might limit CMPs’ use of the results from evidence-based synopses, as such evidence was considered to be incompatible with traditional practice paradigm.

#### Design quality and packaging

The main strength of evidence-based synopses was to promote CMPs’ evidence uptake via a straightforward and concise presentation of evidence-based information. CMPs expressed positive views on clear presentations of quantitative study findings, facilitating easy interpretation: “*Clear details provided in the synopses allowed me to determine whether the treatment is useful to apply on my patients efficiently.” [017].*

Nonetheless, CMPs claimed that numbers alone were not enough for translating evidence into practice. This was because the synopses were not accompanied by commentaries which would address the clinical applicability of findings: *“It would be better to invite CM experts to give contextual or applicability comments for each synopsis. These comments should focus on how or when to apply the evidence-based treatments on different patients, typically with different co-morbidities.” [018].*

### Inner setting

#### Readiness for implementation: available resources

While CMPs who have relevant training were able to interpret results reported in the synopses efficiently, those without training were much less confident. They also did not regard EBHC education resources as readily available after graduation: *“Only fundamental EBHC topics are covered in the undergraduate degree curriculum. Postgraduate training is not provided regularly in the workplace.” [004]* The lack of education opportunities was particularly acute among those working in the private sector, due to conflicting schedules: *“Even though some training workshops are taking place in the evening, CMPs working in private clinics still cannot attend as they have longer working hours and a fluctuating work schedule.” [014]*Limited consultation time restricted them to search and apply synopses in routine practice: *“I only have a few minutes for each consultation, so I may not be able to retrieve and apply evidence in my busy clinical practice.” [005]* This presented another resource limitation for implementation.

#### Readiness for implementation: access to knowledge and information

In this study, an open-access synopses website enabled CMPs to search synopses easily without any charges. This was particularly welcomed among CMPs who work in the private sector: *“Working outside of the public or academic sector, I do not have free access rights to medical databases, and therefore new knowledge from research studies is hardly accessible for me. An open-access synopses website will definitely attract me to look at synopses results and subsequently inform my future routine practice.” [004].*

#### Networks and communication

In semi-public CM clinics, regular peer-sharing sessions and shadowing programs provided opportunities for CMPs to discuss how and when effective interventions reported in synopses might be applied in different clinical scenarios: *“We do have regular peer-sharing sessions at our semi-public CM clinic, offering an opportunity to discuss the applicability of synopses findings with other CMPs. Also, senior CMPs will share their clinical experience with me during shadowing programs, which is another opportunity to seek expert input on synopses applicability.” [016].*

### Outer setting

#### External policy and incentives

Some synopses reported summary of positive effects on CM treatments and they affirmed that CM was evidence-based. Therefore, some CMPs thought that *“Wider dissemination of synopses might promote increment of government subsidy to semi-public clinics.” [020]* Such evidence might even be adopted for creating clinical guidelines and protocol, and hence improving quality of care: *“As we usually work in a rotating schedule in the semi-public clinics. Due to government subsidy policy, patients would consult a different CMP at each follow-up. In order to ensure continuity of care, effective interventions described in the synopses may be used for creating a standardized treatment protocol, such that different CMPs would have a consensus on best practice and ensure continuity of care.” [016].*

Positive evidence reported in synopses might also serve as a basis for promoting inter-professional collaboration with conventional clinicians: *“Research evidence has proved that our CM interventions work. These evidence-based synopses are very meaningful in showing other healthcare professionals the benefits of collaborating with us.” [011].*

#### Patient needs and resources

Data on effectiveness and safety reported in synopses were also regarded as a source of reference in the provision of evidence-based information for patients and family members: *“Some patients are interested in detailed explanations on the treatment prescribed, especially regarding effectiveness. Quantitative data reported in the synopses enable me to explain these to my patients in an objective manner.” [023]*

## Discussion

We identified key barriers and facilitators to using results from clinical evidence synopses in routine practice among CMPs trained in Hong Kong after reunification with China in 1997. The barriers and facilitators were mapped onto constructs in four of the CFIR domains. Barriers were i) CMPs’ perceived difficulties in applying complex evidence in decision-making (intervention characteristics domain) and ii) inadequate training and limited consultation time (inner setting domain). Facilitators included i) availability of publicly accessible and user-friendly synopses, and ii) formation of community of evidence-based practice among CMPs with input from key opinion leaders (inner setting domain); as well as iii) opportunity for interprofessional collaborations with conventional healthcare providers, and iv) patients’ demand for evidence-based clinical advice (outer setting domain). Besides, i) CMPs’ knowledge and beliefs in EBHC (characteristics of individuals), ii) presentations of evidence-based information in the synopses, and iii) clinical decision making as influenced by quality of evidence reported (intervention characteristics domain) acted as both barriers and facilitators. These factors are likely interdependent. For example, inadequate training and consultation time might negatively affect CMPs’ knowledge level and beliefs in EBHC, which would increase the perceived difficulties in applying complex evidence within limited time.

Some barriers identified in our study concurs with previous studies on the use of clinical evidence in routine practice among different healthcare professionals. For instance, physiotherapists and nurses regarded limited resources, in terms of training opportunities and consultation time, as barriers to their adoption of evidence in routine practice [20, 21]. TCIM practitioners, including naturopaths, chiropractors and traditional Korean medicine practitioners, also showed concerns about the deviation from their traditional practice paradigm when evidence is applied in decision-making [22–24]. It is unsurprising that lack of knowledge in interpreting quality of evidence might inhibit the use of evidence in TCIM practice, especially among practitioners who have inadequate research experiences [25].

On the other hand, results of this study showed that evidence was often regarded as a “last resort” to consult, or when the practitioner faces unfamiliar clinical problem. Amongst CMPs in our study, the usefulness of evidence in informing practice is less important than their roles in gaining legitimacy from the health system and conventional healthcare providers, as well as trust from patients [26, 27]. However, use of evidence seems to be more prevalent among CMPs with relevant EBHC training. This behavior may spread via professional socialization and supervision processes in healthcare practice. In the following, we will discuss how preliminary implementation strategies may be tailored to facilitate evidence use in Hong Kong CMPs’ practice.

There are some possible strategies targeting CMPs. Existing evidence suggests that education sessions might promote evidence uptake but the effect is expected to be small [28]. The use of interactive strategies might enhance educational outcomes, but are probably insufficient for changing practice behavior [28]. This is illustrated in the evaluation result of a 3-day interactive EBHC workshop for Hong Kong CMPs [10]. Results of the workshop demonstrated that improvements were mostly confined to attitude and knowledge [10]. Our study findings suggested that peer-sharing in journal club formats might promote collaborative learning and subsequent evidence uptake, which is consistent with current experiences [29]. In semi-public CM clinics, availability of senior CMPs as key opinion leaders may also promote evidence-based practice [30]. Their clinical expertise would allow them to comment on the applicability of evidence reported in the synopses. This process can be instrumental in spreading evidence use in the profession. Finally, to tackle the perceived difficulties in accessing relevant clinical evidence from synopses, professional bodies and academia might consider establishing relevant evidence refinery service. Such service would eventually contribute to upgrading of professional standard and quality. In longer term, artificial intelligence-based linkage between evidence-based synopses and electronic health record can be created. The “on the spot” application of evidence can hence be facilitated during the routine consultation process [31].

A number of potential strategies targeting other stakeholders are also suggested. Our results suggested that CMPs might actively adopt evidence in response to patients’ request. This hints the potential usefulness of patient–mediated interventions for promoting evidence uptake. Existing evidence suggests that patient education interventions may enhance healthcare professionals’ adherence to evidence-based clinical practice [32]. In these interventions, patients are empowered with enhanced self-management skills, which might translate to higher motivation for active involvement in decision-making. Subsequently, CMPs may response to these requests by adopting evidence-based recommendations [33].

CMPs’ current use of evidence in gaining legitimacy from conventional healthcare providers may well be exploited for improving practice via interprofessional collaboration. A recent SR reported that interprofessional interactions was effective in encouraging adherence to evidence-based practice [34]. In health systems like Hong Kong, where conventional medicine is dominant, CMPs are seeking opportunities in collaborating with medical professionals. This interprofessional collaboration would foster integration of CM into the health systems [35]. Besides, such collaboration often entails the development of evidence-based shared care protocol [36]. Therefore, CMPs may be more inclined to practice in accordance to published evidence, with the price of giving up some autonomy in practicing the traditional individualized treatment approach [37]. However, this compromise is likely to be of limited scope. According to the studies conducted in the US and UK, CMPs from both health systems expressed concerns on deviation from CM tradition of individualized syndrome differentiation when adopting evidence recommending a standardized treatment [9, 38]. A recent example on this debate is reflected in Chinese national CM guidelines for COVID-19, of which the advocates of evidence-based CM recommends standardized treatment while other CMPs promotes the use of an individualized approach [39]. A reform on CM diagnostic research is required to address this complex paradigm incompatibility [40]. The recently established ICD-11 Traditional Medicine offers a starting point for this long endeavor [41].

Future researchers may consider tailoring the aforementioned suggestions into practical implementation strategies [42, 43]. The use of CFIR in this study would facilitate selection of possible implementation strategies by matching current findings to the Expert Recommendations for Implementating Change (ERIC) [43, 44]. ERIC is a comprehensive catalogue of implementation strategies [43, 44]. By utilizing the CFIR-ERIC Matching Tool [44], some of the preliminary observations may be enriched into innovative implementation strategies. Prior to promongulation, consensus among different stakeholders on the applicability of these implementation strategies should be evaluated using Delphi survey [45]. Through this process, stakeholders-endorsed implementation strategies would be contextualized to address local needs.

We found the structure and contents of CFIR relevant for our study. This would facilitate the categorization of barriers and facilitators to using results from evidence-based synopses. Our findings also demonstrated the importance of applying such a multifactorial approach to investigate the interdependency and interactions between various determinants in different CFIR domains. Furthermore, we achieved a 100% response rate to invitations in this study. This might be because the participating CMPs were recruited via two investigators’ alumni networks (VCHC and JVHT) in two different Schools of Chinese Medicine in Hong Kong. They would have a higher level of trust in the research team [46]. The participants were informed that their contributions would be useful to inform local education and research needs. Thus, they might be more eager to participate in this study. To avoid the perception of coercion, professional background of the research team has been made explicit to the participants prior to obtaining informed consents [47]. During the recruitment process, the research team has also emphasized to potential study participants that their participations were voluntary and they had right to withdraw at any time [46].

Nonetheless, there are some limitations in this study. Firstly, since this study was conducted in Hong Kong, findings might not be generalizable to other health systems. Barriers and facilitators to using results from evidence-based synopses in routine practice identified may not be an exhaustive list of all possible determinants. That said, Hong Kong’s experience in the past two decades may still offer other health systems insights on how to encourage uptake of current clinical evidence in TCIM practice, especially those which are starting to develop TCIM recently. Secondly, since the EBHC approach is the officially endorsed direction for promoting integraton of CM into local health system, social desirability bias may have skewed views on EBHC toward the positive side [48]. CMPs who were hesititant to the EBHC approach might not be willing to express their viewpoints truthfully. As an atttempt to minimize such bias during data collection, the research team has provided assurances by reminding the participtants about confidentiality of their contributions [48]. Lastly, only CMPs were interviewed in our study. The influence of patients, funders, administrators and other healthcare professionals on evidence use in CM remains unexplored in the current study [49].

In terms of implications, policy makers may consider developing peer learning community among CMPs for promoting EBHC skills acquisition. This can be supplemented by providing up-to-date clinical evidence synopses to enhance evidence implementation in CM. Input from key opinion leaders may facilitate critical interpretation of evidence applicability. The need to respond to patients’ expectations, and interprofessional collaboration opportunities with conventional healthcare providers may also encourage evidence uptake in CM practice. Development of refined implementation strategies in Hong Kong, or in other health systems, would require further input from various key stakeholders, including but not limited to patients, funders, administrators, TCIM and conventional clinicians.

## Conclusions

This CFIR-based qualitative study investigated how the WHO recommendation of promoting evidence use in routine practice was perceived by CMPs trained in Hong Kong after reunification with China in 1997. The identified barriers to applying clinical evidence synopses in routine practice included i) CMPs’ perceived difficulties in applying complex evidence in decision-making and ii) inadequate training and limited consultation time. The facilitators were i) availability of publicly accessible and user-friendly synopses, ii) formation of community of evidence-based practice among CMPs with input from key opinion leaders, iii) opportunity for interprofessional collaborations with conventional healthcare providers, and iv) patients’ demand for evidence-based clinical advice. Besides, i) CMPs’ knowledge and beliefs in EBHC, ii) presentations of evidence-based information in the synopses and iii) clinical decision making as influenced by quality of evidence reported acted as both barriers and facilitators. Implications from these findings may inform tailoring of implementation strategies in the future, in the context of a well-developed health system with dominance of conventional medicine.

## Supplementary Information

**Additional file 1.**

**Additional file 2.**

## Data Availability

The datasets used and analysed during the current study are available from the corresponding author on reasonable request.

## Abbreviations

CM: Chinese Medicine
CMP: Chinese Medicine practitioner
CFIR: Consolidated Framework for Implementation Research
EBHC: Evidence-based healthcare
ERIC: Expert Recommendations for Implementating Change
RCT: Randomized controlled trial
SR: Systematic review
TCIM: Traditional, complementary and integrative medicine
WHO: World Health Organization
