# Supplementary material for: Barriers and facilitators to promoting evidence uptake in Chinese medicine: a qualitative study in Hong Kong
Source: BMC Complement Med Ther. 2021 Jul 15;21:200. doi: 10.1186/s12906-021-03372-5 (PMC8280573; doi:10.1186/s12906-021-03372-5)
Supplement: Supplementary file 1 — Additional file 1. [file 12906_2021_3372_MOESM1_ESM.docx]

**Title Page**

**Title:** Barriers and facilitators to promoting evidence uptake in Chinese medicine: A qualitative study in Hong Kong

**Full names and Institutional Addresses of Authors​​​​​​​:**

**Charlene Hoi Lam Wong**: Rm 509, Jockey Club School of Public Health and Primary Care, Prince of Wales Hospital, Shatin, Hong Kong.

**Jeffrey Van Ho Tse**: Rm 509, Jockey Club School of Public Health and Primary Care, Prince of Wales Hospital, Shatin, Hong Kong.

**Per Nilsen**: Department of Health, Building 511-001, Entrance 76, plan 13, Campus US, Medicine and Caring Sciences, Linköping University, 58183 LINKÖPING, Linköping, Sweden.

**Leonard Ho**: School of Chinese Medicine, LG02, Lee Wai Chun Building, Chung Chi College, The Chinese University of Hong Kong, Shatin, Hong Kong.

**Irene Xin Yin Wu**: Rm 527, 5/F, Department of Epidemiology and Health Statistics, Xiangya School of Public Health, Central South University, 238 Shangmayuanling Alley, Kaifu District, Changsha, China.

**Vincent Chi Ho Chung**: Rm 413, Jockey Club School of Public Health and Primary Care, Prince of Wales Hospital, Shatin, Hong Kong.

**Corresponding Author:**

Irene Xin Yin Wu, Email: irenexywu@csu.edu.cn,

Telephone: +86 0731 84805414, Address: Rm 527, 5/F, Department of Epidemiology and Health Statistics, Xiangya School of Public Health, Central South University, 238 Shangmayuanling Alley, Kaifu District, Changsha, China.

**Additional file 1. English and Chinese versions of the four synopses presented to the Chinese medicine practitioners prior to interviews**

**Is individualized Chinese herbal formula effective in treating oligomenorrhoea and amenorrhoea among female with polycystic ovary syndrome as compared to standardized Chinese herbal formula?**

**(Date of publication of randomized controlled trial: February 2017)**

| **Design** | Pragmatic randomized controlled trial (RCT). |
| --- | --- |
| **Participants** | 40 women presented with oligomenorrhoea or amenorrhoea with a diagnosis of polycystic ovary syndrome consistent with the Rotterdam criteria (age range: 18 to 44 years). |
| **Intervention** | Individualized Chinese herbal medicine (CHM), 16 g daily taken orally as a decoction. Duration of treatment lasted for 6 months. The treatment was given by a practitioner who was registered with a professional CHM organization in the UK and has been practicing for at least 5 years. At baseline, weeks 4, 8 and 12, participants were asked to meet the practitioner. An individualized prescription was formulated by practitioner for every participant at each visit and practitioner might prescribe treatment as usual from a range of 270 different CHMs. |
| **Comparator** | Comparison: Individualized CHM versus standardized CHM. The standardized prescription was fixed, which contained 14 CHMs derived from expert consensus process. Duration of treatment lasted for 6 months, with CHMs taken orally as decoction. |
| **Major Outcomes** | Outcome 1: Change in mean menstrual rate at 6 months;  Outcome 2: Hirsutism as measured by the change in modified Ferriman-Gallwey (mFG) score at 6 months;  Outcome 3: Change in waist-to-hip ratio (WHR) at 6 months;  Outcome 4: Change in quality of life as measured by Polycystic Ovary Syndrome (PCOS) total scores at 6 months. |
| **Settings** | The trial was conducted in outpatient settings. |

**Comparison Individualized CHM versus standardized CHM**

**Main results**

Compared to standardized CHM, individualized CHM did not show significant improvement in the mean menstrual rate (mean differences (MD): 0.10, 95% CI: -0.07 to 0.26), mFG scores (p= 0.09) and PCOS total scores (p= 0.33) at 6 months among women with polycystic ovary syndrome. However, individualized CHM showed significant reduction in WHR when compared to standardized CHM (p=0.02). Both standardized and individualized CHM conferred benefits to patients across all outcomes.


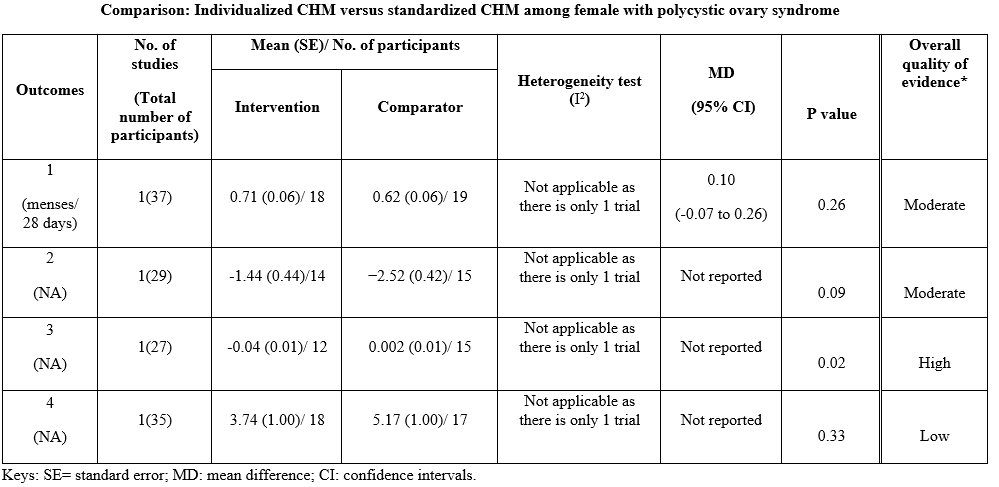


**Conclusion**

**Benefits**

This study showed that both individualized and standardized CHM can improve outcomes of patients with polycystic ovary syndrome. There were no significant differences on the mean menstrual rate, mFG scores and PCOS total scores at 6 months between individualized CHM and standardized CHM groups. However, individualized CHM significantly reduced WHR when compared to standardized CHM.

For outcomes 1 and 2, the overall quality of evidence is moderate. Further research is fairly likely to have an important impact on our confidence in this estimate of effect.

For outcome 3, the overall quality of evidence is high. Further research is unlikely to have an important impact on our confidence in this estimate of effect.

For outcome 4, the overall quality of evidence is low. Further research is likely to have an important impact on our confidence in this estimate of effect.

**Harms**

Two serious adverse events were reported. One patient in individualized CHM group was diagnosed with nodular goitre and one in standardized CHM group experienced a leg fracture. Both events were confirmed to be not related to study treatments.

Adverse events were minor with 9 reactions reported among six patients in standardized CHM group and four in individualized CHM group. The 9 events were assessed as mild, expected, not serious and having a reasonable causal relationship with treatment. The most commonly reported symptoms are gastrointestinal symptoms, including bloating, nausea, loose stools and vomiting while the remaining consist of tiredness, skin breakout, ovulation pain and headache.

**Link to original article**

<https://www.ncbi.nlm.nih.gov/pmc/articles/PMC5293993/>

**The synopsis is based on the following article:**

Lai L, Flower A, Prescott P, Wing T, Moore M, Lewith G. Standardised versus individualised multiherb Chinese herbal medicine for oligomenorrhoea and amenorrhoea in polycystic ovary syndrome: a randomised feasibility and pilot study in the UK. BMJ open. 2017 Feb 1;7(2):e011709.

*** Interpretation of quality assessment results:**

• Very low: Further research is most likely to have an important impact on our confidence in this estimate of effect.

• Low: Further research is likely to have an important impact on our confidence in this estimate of effect.

• Moderate: Further research is fairly likely to have an important impact on our confidence in this estimate of effect.

• High: Further research is unlikely to have an important impact on our confidence in this estimate of effect.

• Very high: Further research is most unlikely to have an important impact on our confidence in this estimate of effect.

Details of assessment method can be found at Chung VC, Wu XY, Ziea ET, Ng BF, Wong SY, Wu JC. Assessing internal validity of clinical evidence on effectiveness of CHinese and integrative medicine: Proposed framework for a CHinese and Integrative Medicine Evidence RAting System (CHIMERAS). European Journal of Integrative Medicine. 2015 Aug 31;7(4):332-41.

**與標準方相比，辨證論治對改善女性多囊卵巢綜合症中月經次數過少和停經的情況有效嗎？**

**(隨機對照試驗發佈日期：二零一七年二月)**

| **研究設計** | 務實隨機對照試驗。 |
| --- | --- |
| **參加者** | 40名根據鹿特丹標準診斷為多囊卵巢綜合症並出現月經次數過少和停經情況的女性病人（年齡範圍：18至44歲）。 |
| **治療組** | 辨證論治組的病人會在6個月期間接受一位有5年以上臨床經驗的中醫師治療。所有醫師均已向相關的英國中醫師公會註冊。在初診、第4、8和12週，病人與醫師見面，而中醫師可按中醫診斷從270種單味沖劑中選擇用藥。每兩次覆診之間，用方不變。其中最常用的20味中藥為: 川芎、醋香附、柴胡、白芍、炙甘草、白花蛇舌草、當歸、熟地黃、牡丹皮、赤芍、生甘草、生地黃、桂枝、枳殼、茯苓、陳皮、延胡索、當歸尾、黃連和梔子。 |
| **對照組** | 對照：辨證論治對比標準方  標準方組的病人每天服用以下中藥沖劑6個月: 白芍 15g、柴胡 9g、陳皮9g、川芎9g、當歸尾9g、炙甘草6g、杞子9g、桂枝9g、紅花9g、桃仁9g、菟絲子12g、益母草15g、枳殼9g和醋香附12g (此方劑根據專家共識設計) |
| **主要結果 (詳見下表)** | 結果一：經過6個月治療後，平均月經次數(每28天)的改變；  結果二：經過6個月治療後，使用modified Ferriman-Gallwey (mFG) 量表量度與多毛症有關的分數改變；  結果三：經過6個月治療後，腰臀圍比值(WHR) 的改變;  結果四：經過6個月治療後，使用多囊卵巢綜合症 (PCOS)總分量度的生活素質改變。 |
| **設置** | 此研究在門診進行。 |

**對照 辨證論治對比標準方**

**主要結果**

與標準方組相比，經過6個月治療後，辨證論治組及標準方組都可以有效改善病人的上述4個指標。兩組之間的結果比較如下：辨證論治組未能更有效地增加多囊卵巢綜合症女性的平均月經次數（平均數差異(MD): 0.10, 95% CI: -0.07 至 0.26），減少mFG 分數 (p值= 0.09)及提升生活素質(p值= 0.33)。但是，與標準方組相比，辨證論治組則更有效地減少了腰臀圍比值(p值= 0.02)。


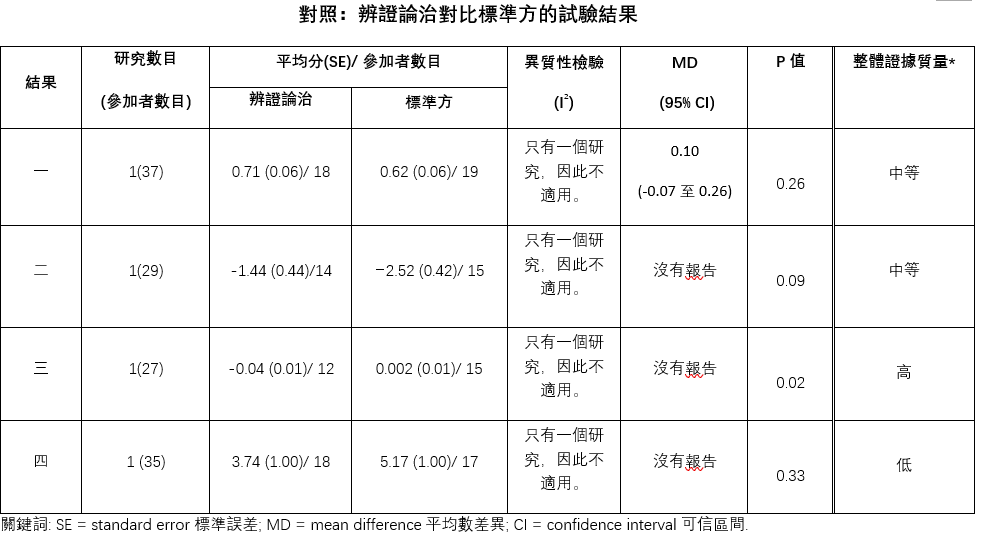


**結論**

**效果**

經過6個月治療後，辨證論治組及標準方組都可以改善女性多囊卵巢綜合症中月經次數過少和停經的情況。與標準方組相比，辨證論治組可以更有效地減少腰臀圍比值。整體證據質量屬於高。進一步的研究不太可能改變我們對效應估計值的可信性。

然而，兩組病人在平均月經次數、mFG 分數及生活素質3個主要結果中的MD於統計學上並沒有顯著的差異。

針對結果一和二，整體證據質量屬於中等。進一步的研究可能改變我們對效應估計值的可信性。

針對結果四，整體證據質量屬於低。進一步的研究很可能改變我們對效應估計值的可信性。

**不良反應和副作用**

研究共報告了兩個嚴重不良反應，辨證論治組有1個病人被診斷為結節性甲狀腺腫，標準方組則有1人腿骨折。兩個個案被確認與研究提供的治療沒有關係。

4個辨證論治組的病人及6個標準方組的病人共出現了9個不良反應。常見的不良反應為腸胃不適的症狀，如: 腹脹，噁心，稀便和嘔吐，而其他則包括疲勞，皮膚乾裂，排卵疼痛，頭痛，均屬預料中與治療有關的輕微副作用。

**原文鏈結**

<https://www.ncbi.nlm.nih.gov/pmc/articles/PMC5293993/>

**本概要源於以下文章:**

Lai L, Flower A, Prescott P, Wing T, Moore M, Lewith G. Standardised versus individualised multiherb Chinese herbal medicine for oligomenorrhoea and amenorrhoea in polycystic ovary syndrome: a randomised feasibility and pilot study in the UK. BMJ open. 2017 Feb 1;7(2):e011709.

***整體證據質量5個等級的含義:**

• 極低: 進一步的研究非常可能改變目前效應估計值的可信性。

• 低: 進一步的研究很可能改變目前效應估計值的可信性。

• 中等: 進一步的研究可能改變目前效應估計值的可信性。

• 高: 進一步的研究不太可能改變目前效應估計值的可信性。

• 極高: 進一步的研究極少可能改變目前效應估計值的可信性。

有關證據質量評估的詳情可參考 Chung VC, Wu XY, Ziea ET, Ng BF, Wong SY, Wu JC. Assessing internal validity of clinical evidence on effectiveness of CHinese and integrative medicine: Proposed framework for a CHinese and Integrative Medicine Evidence RAting System (CHIMERAS). European Journal of Integrative Medicine. 2015 Aug 31;7(4):332-41.

**Is the combination of oseltamivir and Chinese herbal formula**

**maxingshigan-yinqiaosan effective in treating H1N1 Influenza?**

**(Date of publication of randomized controlled trial: August 2011)**

| **Design** | Randomized controlled trial (RCT). |
| --- | --- |
| **Participants** | 410 adults aged 15 to 70 years presented within 72 hours of onset of H1N1 influenza A symptoms (mean age: 19.0 years; 57.1% male). |
| **Intervention** | The combination of oseltamivir and Chinese herbal formula maxingshigan-yinqiaosan. Oral oseltamivir, 75 mg twice daily; maxingshigan–yinqiaosan decoction which was composed of 12 Chinese herbal medicines, 200 mL orally 4 times daily. Duration of treatment lasted for 5 days. |
| **Comparator** | Comparison 1: Oseltamivir plus maxingshigan-yinqiaosan versus no intervention;  Comparison 2: Oseltamivir plus maxingshigan-yinqiaosan versus oseltamivir alone. |
| **Major Outcomes** | Outcome 1: Time from randomization to fever resolution (body temperature ≤37 °C  for 24 hours). |
| **Settings** | The trial was conducted in inpatient settings. |

**Comparison 1 Oseltamivir plus maxingshigan-yinqiaosan versus no intervention**

**Main results**

Compared to no intervention, oseltamivir plus maxingshigan-yinqiaosan significantly reduced the median time to fever resolution by 47% (95% CI: 35% to 56%).


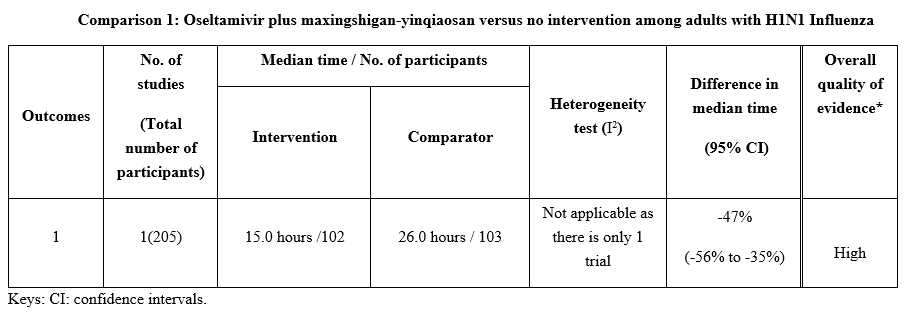


**Comparison 2 Oseltamivir plus maxingshigan-yinqiaosan versus oseltamivir alone**

**Main results**

Compared to oseltamivir alone, the combination of oseltamivir and maxingshigan-yinqiaosan significantly reduced the median time to fever resolution by 19% (95% CI: 0.3% to 34%).


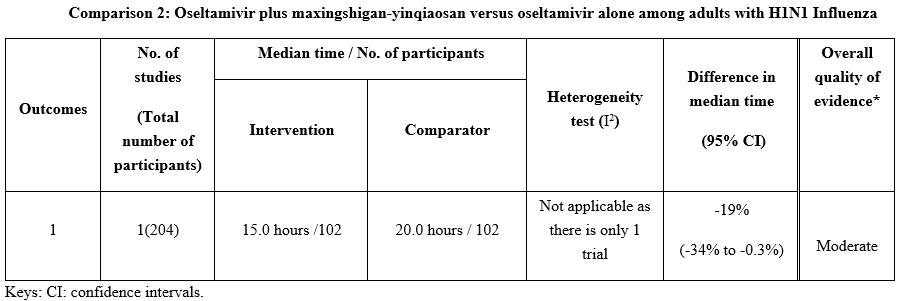


**Conclusion**

**Benefits**

This study showed that the combination of oseltamivir and maxingshigan-yinqiaosan significantly reduced the median time to fever resolution when compared to no intervention and Oseltamivir alone.

For outcome 1 in Comparison 1, the overall quality of evidence is high. Further research is unlikely to have an important impact on our confidence in this estimate of effect.

For outcome 1 in Comparison 2, the overall quality of evidence is moderate. Further research is fairly likely to have an important impact on our confidence in this estimate of effect.

**Harms**

No adverse events were observed in no intervention group, oseltamivir alone group and the combination of oseltamivir and maxingshigan-yinqiaosan group.

**Link to original article**

<https://www.ncbi.nlm.nih.gov/pubmed/21844547>

**The synopsis is based on the following article:**

Wang C, Cao B, Liu QQ, Zou ZQ, Liang ZA, Gu L, Dong JP, Liang LR, Li XW, Hu K, He XS. Oseltamivir Compared With the Chinese Traditional Therapy Maxingshigan–Yinqiaosan in the Treatment of H1N1 InfluenzaA Randomized Trial. Annals of Internal Medicine. 2011 Aug 16;155(4):217-25.

*** Interpretation of quality assessment results:**

• Very low: Further research is most likely to have an important impact on our confidence in this estimate of effect.

• Low: Further research is likely to have an important impact on our confidence in this estimate of effect.

• Moderate: Further research is fairly likely to have an important impact on our confidence in this estimate of effect.

• High: Further research is unlikely to have an important impact on our confidence in this estimate of effect.

• Very high: Further research is most unlikely to have an important impact on our confidence in this estimate of effect.

Details of assessment method can be found at Chung VC, Wu XY, Ziea ET, Ng BF, Wong SY, Wu JC. Assessing internal validity of clinical evidence on effectiveness of CHinese and integrative medicine: Proposed framework for a CHinese and Integrative Medicine Evidence RAting System (CHIMERAS). European Journal of Integrative Medicine. 2015 Aug 31;7(4):332-41.

**奧司他韋(特敏福)加中藥麻杏石甘湯-銀翹散**

**對治療甲型H1N1流感有效嗎？**

**(隨機對照試驗發佈日期：二零一一年八月)**

| **研究設計** | 隨機對照試驗。 |
| --- | --- |
| **參加者** | 410名15至70歲有甲型H1N1流感症狀達72小時的病人（平均年齡：19.0歲，57.1%男性）。 |
| **治療組** | 特敏福加中藥麻杏石甘湯-銀翹散:  口服特敏福(75mg, 兩次/日);  中藥為麻杏石甘湯-銀翹散加減5天，每天4次，用以下中藥水煎成200mL口服: 炙麻黃6g、炒杏仁15g、石膏30g、生甘草10g、知母10g、青蒿15g、銀花15g、黃芩15g、連翹15g、薄荷6g、浙貝母10g和牛蒡子15g。 |
| **對照組** | 對照一：特敏福加中藥麻杏石甘湯-銀翹散對比沒有接受治療；  對照二：特敏福加中藥麻杏石甘湯-銀翹散對比只有特敏福 |
| **主要結果 (詳見下表)** | 結果一：從隨機分組到退燒(體溫持續24小時低於攝氏37度)的時間。 |
| **設置** | 此研究在住院病人中進行。 |

**對照一 特敏福加中藥麻杏石甘湯-銀翹散對比沒有接受治療**

**主要結果**

與沒有接受治療相比，特敏福加中藥麻杏石甘湯-銀翹散將退燒時間的中位數顯著縮短47%(95% CI: 35%至56%)。


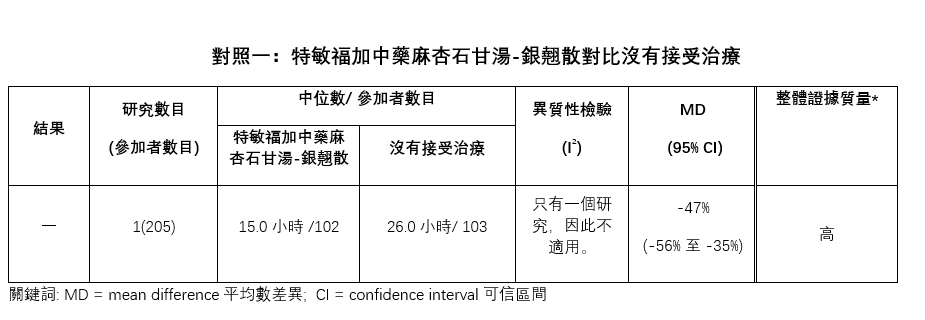


**對照二 特敏福加中藥麻杏石甘湯-銀翹散對比只有特敏福**

**主要結果**

與只有特敏福相比，特敏福加中藥麻杏石甘湯-銀翹散將退燒時間的中位數顯著縮短19%(95% CI: 0.3%至34%)。


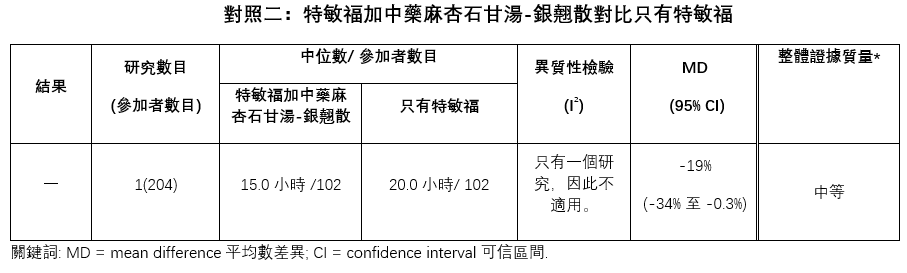


**結論**

**效果**

相比沒有接受治療，特敏福加中藥麻杏石甘湯-銀翹散可顯著地縮短退燒時間。整體證據質量屬於高。進一步的研究不太可能改變我們對效應估計值的可信性。

相比只有特敏福，特敏福加中藥麻杏石甘湯-銀翹散顯著地縮短退燒時間。整體證據質量屬於中等。進一步的研究可能改變我們對效應估計值的可信性。

**不良反應和副作用**

研究指出沒有接受治療組、只有特敏福組及特敏福加中藥麻杏石甘湯-銀翹散組均沒有出現任何不良反應。

**原文鏈結**

<https://www.ncbi.nlm.nih.gov/pubmed/21844547>

**本概要源於以下文章:**

Wang C, Cao B, Liu QQ, Zou ZQ, Liang ZA, Gu L, Dong JP, Liang LR, Li XW, Hu K, He XS. Oseltamivir Compared With the Chinese Traditional Therapy Maxingshigan–Yinqiaosan in the Treatment of H1N1 InfluenzaA Randomized Trial. Annals of Internal Medicine. 2011 Aug 16;155(4):217-25.

***整體證據質量5個等級的含義:**

• 極低: 進一步的研究非常可能改變目前效應估計值的可信性。

• 低: 進一步的研究很可能改變目前效應估計值的可信性。

• 中等: 進一步的研究可能改變目前效應估計值的可信性。

• 高: 進一步的研究不太可能改變目前效應估計值的可信性。

• 極高: 進一步的研究極少可能改變目前效應估計值的可信性。

有關證據質量評估的詳情可參考 Chung VC, Wu XY, Ziea ET, Ng BF, Wong SY, Wu JC. Assessing internal validity of clinical evidence on effectiveness of CHinese and integrative medicine: Proposed framework for a CHinese and Integrative Medicine Evidence RAting System (CHIMERAS). European Journal of Integrative Medicine. 2015 Aug 31;7(4):332-41.

**What is the comparative effectiveness of combining different Chinese herbal medicine and salmeterol and fluticasone propionate (SFP) in treating chronic obstructive pulmonary disease as compared to SFP alone?**

**(Date of publication of systematic review and network meta-analysis: May 2016)**

| **Design** | Systematic review and network meta-analysis (NMA) of 11 randomized controlled trials (RCTs). |
| --- | --- |
| **Participants** | 925 patients with a diagnosis of chronic obstructive pulmonary disease (COPD) (mean age: 65.5 years; gender proportion was not reported by the reviewers). 9 studies applied the Global Initiative for Chronic Obstructive Lung Disease (GOLD) guideline [postbronchodilator FEV1 <80% of the predicted value, with a ratio of FEV1 to forced vital capacity (FVC) <70%] for diagnosis and management of COPD. |
| **Intervention** | The Chinese herbal medicine (CHM) formulations were different for each trial. Among the 11 types of CHM interventions, 7 were herbal decoctions, 1 was prescribed as pills, and the remaining 3 were capsules. SFP were used twice daily. Duration of treatments were at least 12 weeks. |
| **Comparators** | Comparison: Different CHM plus SFP vs SFP alone. |
| **Major Outcomes** | Outcome 1: Change in FEV1, follow-up period varied from 3 months to 1 year;  Outcome 2: Change in health-related quality of life measured by the St George’s Respiratory Questionnaire (SGRQ), follow-up period varied from 3 months to 1 year. |
| **Settings** | 5 trials were conducted in outpatient settings and 5 trials were conducted in both inpatient and outpatient settings. One trial did not specify the study settings. |

**Comparison Different CHM plus SFP vs SFP alone**

**Main results**

From the results of meta-analysis, compared to SFP alone, the combination of CHM and SFP showed significant improvement in FEV1 (pooled weighted mean difference (WMD): 0.20, 95% CI: 0.06 to 0.34) and patients’ quality of life (pooled WMD: -4.99, 95% CI: -7.33 to -2.24) with follow-up period varying from 3 months to 1 year.


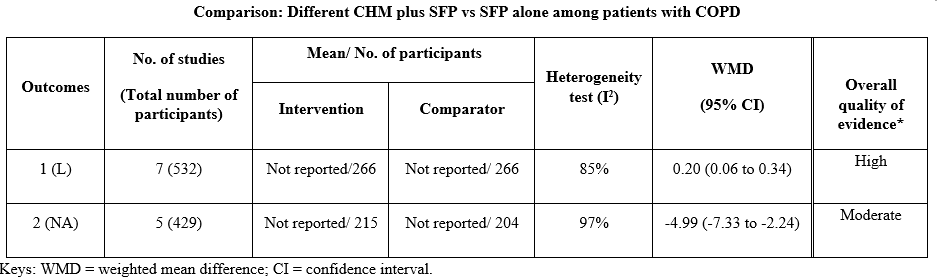


**Results of Network meta-analysis on changes in FEV1**

**Main Results**

Regarding changes in FEV1, there was no statistically significant difference between 7 CHM formulae from the results of NMA. The 7 formulae consisted of similar herbal compositions, such as Cordyceps sinensis (冬蟲夏草), Astragalus membranaceus (黃芪) , Rehmanniae radix preparata (熟地黃), Fructus Schisandrae (五味子), Radix codonopsis (黨參), Gecko (蛤蚧), Root bark of paeonia suffruticosa andr (牡丹皮) and Radix et rhizoma ginseng (人參). The comparative effectiveness rankings of the 8 different treatments were as follow:

| **Ranking of comparative effectiveness** | **Treatments** |
| --- | --- |
| 1st | Jiaweisanao decoction+ SFP |
| 2nd | Baining capsule+ SFP |
| 3rd | Jiajianbufei decoction+ SFP |
| 4th | Baoyuan decoction+ SFP |
| 5th | Shenha capsule+ SFP |
| 6th | Yiqihuoxue decoction+ SFP |
| 7th | SFP only |
| 8th | Jiaweiqiweiduqi decoction+ SFP |

**Results of NMA on changes in QOL**

**Main Results**

Regarding changes in SGRQ scores, there was statistically significant difference between 4 CHM formulae from the results of network meta-analysis. The 4 formulae consisted of similar herbal compositions, such as Astragalus membranaceus (黃芪) , Atractylodis macrocephalae rhizome (白朮), Rehmanniae radix preparata (熟地黃), Radix codonopsis (黨參) and Root bark of paeonia suffruticosa andr (牡丹皮). The comparative effectiveness rankings of the 8 different treatments were as follow:

| **Ranking of comparative effectiveness** | **Treatments** |
| --- | --- |
| 1st | Runfeijianpibushen decoction+ SFP |
| 2nd | Renshenbufei pills+ SFP |
| 3rd | Yiqihuoxue decoction+ SFP |
| 4th | Jiaweiqiweiduqi decoction+ SFP |
| 5th | SFP only |

**Conclusion**

**Benefits**

This study showed that the combination of CHM and SFP significantly improved FEV1 and SGRQ measured quality of life when compared to SFP alone.

For outcome 1, the overall quality of evidence is high. Further research is unlikely to have an important impact on our confidence in this estimate of effect.

For outcomes 2, the overall quality of evidence is moderate. Further research is fairly likely to have an important impact on our confidence in this estimate of effect.

This study indicated that Jiaweisanao decoction+ SFP had a slightly higher probability of being the best treatment in improving patients’ FEV1. However, there was no statistically significant difference between the treatments.

The common herbal compositions are Cordyceps sinensis (冬蟲夏草), Astragalus membranaceus (黃芪) , Rehmanniae radix preparata (熟地黃), Fructus Schisandrae (五味子), Radix codonopsis (黨參), Gecko (蛤蚧), Root bark of paeonia suffruticosa andr (牡丹皮) and Radix et rhizoma ginseng (人參). In addition, Runfeijianpibushen decoction+ SFP had a slightly higher probability of being the best treatment in improving patients’ quality of life. The common herbal compositions are Astragalus membranaceus (黃芪) , Atractylodis macrocephalae rhizome (白朮), Rehmanniae radix preparata (熟地黃), Radix codonopsis (黨參) and Root bark of paeonia suffruticosa andr (牡丹皮).

**Harms**

No adverse events were reported in the included trials.

**Link to original article**

<https://www.ncbi.nlm.nih.gov/pubmed/27196484>

**The synopsis is based on the following article:**

Chung VC, Wu X, Ma PH, Ho RS, Poon SK, Hui DS, Wong SY, Wu JC. Chinese Herbal Medicine and Salmeterol and Fluticasone Propionate for Chronic Obstructive Pulmonary Disease: Systematic Review and Network Meta-Analysis. Medicine. 2016 May;95(20).

*** Interpretation of quality assessment results:**

• Very low: Further research is most likely to have an important impact on our confidence in this estimate of effect.

• Low: Further research is likely to have an important impact on our confidence in this estimate of effect.

• Moderate: Further research is fairly likely to have an important impact on our confidence in this estimate of effect.

• High: Further research is unlikely to have an important impact on our confidence in this estimate of effect.

• Very high: Further research is most unlikely to have an important impact on our confidence in this estimate of effect.

Details of assessment method can be found at Chung VC, Wu XY, Ziea ET, Ng BF, Wong SY, Wu JC. Assessing internal validity of clinical evidence on effectiveness of CHinese and integrative medicine: Proposed framework for a CHinese and Integrative Medicine Evidence RAting System (CHIMERAS). European Journal of Integrative Medicine. 2015 Aug 31;7(4):332-41.

**不同中藥方劑加沙美特羅氟替卡松對比只用沙美特羅氟替卡松(SFP)治療慢性阻塞性肺病的相對有效性**

**(系統性文獻回顧及網絡薈萃分析發佈日期：二零一六年五月)**

| **研究設計** | 11個隨機對照試驗的系統性文獻回顧及網絡薈萃分析。 |
| --- | --- |
| **參加者** | 925名根據全球慢性阻塞性肺病倡議（GOLD）的準則診斷為慢性阻塞性肺病患者 (平均年齡: 65.5歲，研究沒有報告性別比例)，GOLD的準則為使用支氣管擴張劑後的第一秒吐氣量(postbronchodilator FEV_1_) 低於預設值的80%，而FEV_1_/ FVC 的比例< 70%。 |
| **治療組** | 11個不同的中藥方劑，當中7個為中藥湯劑，1個為藥丸，3個為膠囊; 加支氣管擴張劑沙美特羅氟替卡松(Salmeterol and fluticasone propionate, SFP) 兩次/日; 療程為期12週或以上。 |
| **對照組** | 對照：不同中藥方劑加SFP對比只用SFP |
| **主要結果**  **(詳見下表)** | 結果一：隨訪3個月至1年的FEV_1_改變；  結果二：隨訪3個月至1年以聖喬治呼吸問卷(St George’s Respiratory Questionnaire, SGRQ)量度的生活質素改變。 |
| **設置** | 5個試驗均在住院內進行，5個在門診及住院內進行，1個則沒有報告設置詳情。 |

**對照 不同中藥方劑加SFP對比只用SFP**

**主要結果**

**一般薈萃分析的結果**

與只用SFP相比，不同中藥方劑加SFP更有效地改善隨訪3個月至1年的FEV_1_ (合併加權均數差(WMD): 0.20, 95% CI: 0.06 至 0.34) 及提升患者隨訪3個月至1年的生活質素(合併WMD: -4.99, 95% CI: -7.33至 -2.24)。


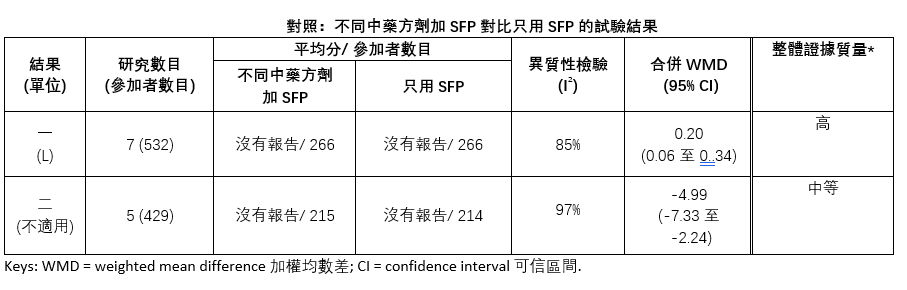


**網絡薈萃分析的結果**

有關FEV_1_改變，網絡薈萃分析未能發現7個中藥方劑的療效之間有統計學差異。在這7個中藥方劑中，最常用的中藥為冬蟲夏草、黃芪、熟地黃、五味子、黨參、蛤蚧、牡丹皮和人參。而8個不同治療方案的相對療效排名如下:

| **排行** | **治療方案** |
| --- | --- |
| 一 | 加味三拗湯+ SFP |
| 二 | 百令膠囊+ SFP |
| 三 | 加減補肺湯+ SFP |
| 四 | 保元湯+ SFP |
| 五 | 參蛤膠囊+ SFP |
| 六 | 益氣活血湯+ SFP |
| 七 | 只有SFP |
| 八 | 加味七味都氣湯+ SFP |

有關SGRQ生活質素改變，網絡薈萃分析發現4個中藥方劑之間有統計學差異，其中潤肺健脾補腎湯加SFP及人參補肺丸加SFP比其他治療方案可更有效地提升患者的生活質素。在這4個中藥方劑中，最常用的中藥為黃芪、白朮、熟地黃、黨參及牡丹皮。而5個不同治療方案的相對療效排名如下:

| **排行** | **治療方案** |
| --- | --- |
| 一 | 潤肺健脾補腎湯+ SFP |
| 二 | 人參補肺丸+SFP |
| 三 | 益氣活血湯+SFP |
| 四 | 加味七味都氣湯+ SFP |
| 五 | 只有SFP |

**結論**

**效果**

與只有SFP相比，不同中藥方劑加SFP更有效地改善隨訪3個月至1年的FEV_1_。整體證據質量屬於高。進一步的研究不太可能改變我們對效應估計值的可信性。

此外，與只有SFP相比，不同中藥方劑加SFP更有效地提升患者隨訪3個月至1年的生活質素。整體證據質量屬於中等。進一步的研究可能改變我們對效應估計值的可信性。

相對而言，加味三拗湯+ SFP可能為最有效改善患者FEV_1_的治療方案，但其分別並無統計學意義。最常用的中藥為冬蟲夏草、黃芪、熟地黃、五味子、黨參、蛤蚧、牡丹皮和人參。

相對而言，潤肺健脾補腎湯+ SFP可能為最有效提升他們生活質素的治療方案，最常用的中藥為黃芪、白朮、熟地黃、黨參及牡丹皮。

**不良反應和副作用**

研究納入的試驗沒有報告任何不良反應。

**原文鏈結**

<https://www.ncbi.nlm.nih.gov/pubmed/27196484>

**本概要源於以下文章:**

Chung VC, Wu X, Ma PH, Ho RS, Poon SK, Hui DS, Wong SY, Wu JC. Chinese Herbal Medicine and Salmeterol and Fluticasone Propionate for Chronic Obstructive Pulmonary Disease: Systematic Review and Network Meta-Analysis. Medicine. 2016 May;95(20).

***整體證據質量5個等級的含義:**

• 極低: 進一步的研究非常可能改變目前效應估計值的可信性。

• 低: 進一步的研究很可能改變目前效應估計值的可信性。

• 中等: 進一步的研究可能改變目前效應估計值的可信性。

• 高: 進一步的研究不太可能改變目前效應估計值的可信性。

• 極高: 進一步的研究極少可能改變目前效應估計值的可信性。

有關證據質量評估的詳情可參考 Chung VC, Wu XY, Ziea ET, Ng BF, Wong SY, Wu JC. Assessing internal validity of clinical evidence on effectiveness of CHinese and integrative medicine: Proposed framework for a CHinese and Integrative Medicine Evidence RAting System (CHIMERAS). European Journal of Integrative Medicine. 2015 Aug 31;7(4):332-41.

**What is the comparative effectiveness of using acupuncture and related therapies as add-on or alternative to prokinetics for functional dyspepsia?**

**(Date of publication of systematic review and network meta-analysis: September 2017)**

| **Design** | Systematic review and network meta-analysis (NMA) of 22 randomized controlled trials (RCTs). |
| --- | --- |
| **Participants** | 1727 patients with a diagnosis of functional dyspepsia (FD) (age range: 17 to 70 years; gender proportion was not reported by the reviewers). Respectively eleven, six and three studies applied the Rome III, Rome II and Rome I criteria. Two studies followed other FD diagnostic criteria which were determined by the authors. |
| **Intervention** | Acupuncture and related therapies or acupuncture and related therapies on top of prokinetics.  Four different forms of acupuncture and related therapies included i) manual acupuncture, ii) manual acupuncture plus moxibustion, iii) moxibustion and iv) electroacupuncture.  Three types of combination therapies included i) manual acupuncture plus clebopride, ii) manual acupuncture plus mosapride, and iii) manual acupuncture plus moxibustion on top of domperidone.  Duration of treatments ranged from two to four weeks. |
| **Comparators** | Comparison 1: Manual acupuncture vs domperidone;  Comparison 2: Manual acupuncture vs itopride;  Comparison 3: Different forms of acupuncture and related therapies plus prokinetics vs prokinetics alone  Four types of prokinetics included i) domperidone, ii) itopride, iii) mosapride and iv) clebopride.  Duration of treatments ranged from two to four weeks. |
| **Major Outcomes** | Outcome 1: Patient reported global FD symptom improvement at the end of the study. |
| **Settings** | The reviewers did not state whether the study was conducted in in-patient or out-patient settings. |

**Comparison 1 Manual acupuncture vs domperidone**

**Main results**

From the results of meta-analysis, when compared to domperidone, manual acupuncture was more effective in alleviating global FD symptom (pooled relative risk (RR): 1.21, 95%CI: 1.10, 1.33).


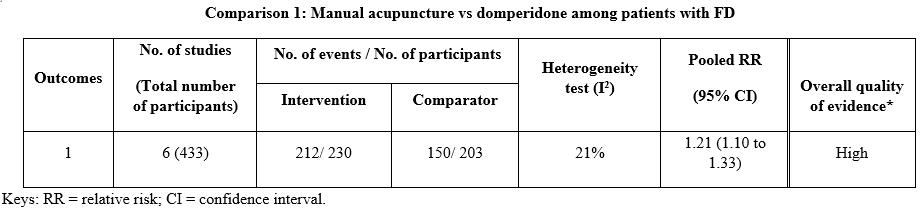


**Comparison 2 Manual acupuncture vs itopride**

**Main results**

From the results of meta-analysis, manual acupuncture was more effective than itopride in relieving global FD symptom (pooled RR: 1.30, 95%CI: 1.11, 1.52).


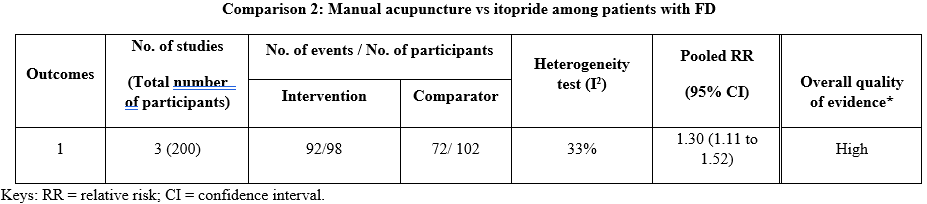


**Comparison 3 Different forms of acupuncture and related therapies plus prokinetics vs prokinetics alone**

**Main Results**

From the results of NMA, there were statistically significant differences in patient reported global FD symptom improvement between 11 interventions which included different forms of acupuncture and related therapies, used alone or as an add-on to prokinetics. The comparative effectiveness rankings of the 11 different interventions were as follow:

| **Ranking of comparative effectiveness** | **Treatments** |
| --- | --- |
| 1st | Manual acupuncture plus clebopride |
| 2nd | Manual acupuncture plus moxibustion on top of domperidone |
| 3rd | Clebopride |
| 4th | Manual acupuncture |
| 5th | Moxibustion |
| 6th | Manual acupuncture plus mosapride |
| 7th | Electroacupuncture |
| 8th | Manual acupuncture plus moxibustion |
| 9th | Domperidone |
| 10th | Mosapride |
| 11th | Itopride |

**Conclusion**

**Benefits**

This study showed that manual acupuncture was more effective in alleviating global FD symptom when compared to domperidone and itopride.

For outcome 1 in comparisons 1 and 2, the overall quality of evidence is high. Further research is unlikely to have an important impact on our confidence in this estimate of effect.

This study indicated that the combination of manual acupuncture and clebopride has the highest probability being the best treatment in improving global FD symptom.

**Harms**

Two studies reported adverse events and they were of minor and transient nature. Ecchymosis (n=4) at acupuncture points were reported after receiving manual acupuncture plus moxibustion, while rash (n = 1) and constipation (n = 2) were reported in the domperidone group. Minor nausea, increased defecation frequency and stomach rumble cases were reported in both mosapride group and the combined manual acupuncture and mosapride group.

**Link to original article**

<https://www.ncbi.nlm.nih.gov/pubmed/28871092/>

**The synopsis is based on the following article:**

Ho RS, Chung VC, Wong CH, Wu JC, Wong SY, Wu IX. Acupuncture and related therapies used as add-on or alternative to prokinetics for functional dyspepsia: overview of systematic reviews and network meta-analysis. Scientific reports. 2017;7(1): 10320.

*** Interpretation of quality assessment results:**

• Very low: Further research is most likely to have an important impact on our confidence in this estimate of effect.

• Low: Further research is likely to have an important impact on our confidence in this estimate of effect.

• Moderate: Further research is fairly likely to have an important impact on our confidence in this estimate of effect.

• High: Further research is unlikely to have an important impact on our confidence in this estimate of effect.

• Very high: Further research is most unlikely to have an important impact on our confidence in this estimate of effect.

Details of assessment method can be found at Chung VC, Wu XY, Ziea ET, Ng BF, Wong SY, Wu JC. Assessing internal validity of clinical evidence on effectiveness of CHinese and integrative medicine: Proposed framework for a CHinese and Integrative Medicine Evidence RAting System (CHIMERAS). European Journal of Integrative Medicine. 2015 Aug 31;7(4):332-41.

**不同針灸方案聯合或代替胃腸動力藥治功能性消化不良的相對有效性**

**(系統性文獻回顧及網絡薈萃分析發佈日期：二零一七年九月)**

| **研究設計** | 22個隨機對照試驗的系統性文獻回顧概述及網絡薈萃分析。 |
| --- | --- |
| **參加者** | 1727名以羅馬標準 或 其他標準診斷的功能性消化不良患者 (年齡範圍: 17至70歲;研究沒有報告性別比例)。 |
| **治療組** | 針灸及相關治療或針灸及相關治療+胃腸動力藥。  四種針灸及相關治療方法包括針灸、艾灸、針灸+艾灸及電針灸。  三種聯合治療包括針灸+氯波必利(clebopride)或莫沙必利(mosapride)及針灸+艾灸+多潘立酮(domperidone)。  治療期間為2至4星期。 |
| **對照組** | 對照一：針灸對比多潘立酮 ;  對照二：針灸對比伊托必利(itopride)；  對照三 : 不同針灸方案+胃腸動力藥對比只用胃腸動力藥。  四種口服胃腸動力藥包括氯波必利、莫沙必利、多潘立酮及伊托必利。治療期間為2至4星期。 |
| **主要結果** | 結果一: 治療後功能性消化不良整體症狀情況的改變。 |
| **設置** | 研究者沒有報告試驗在門診或住院內進行。 |

**對照一 針灸對比多潘立酮**

**主要結果**

**一般薈萃分析的結果**

與多潘立酮相比, 針灸可以更有效地改善功能性消化不良整體症狀 (合併RR: 1.21, 95%CI: 1.10 to 1.33) 。


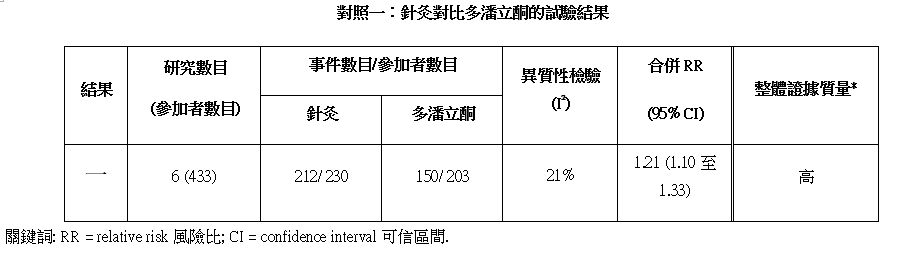


**對照二 針灸對比伊托必利**

**主要結果**

**一般薈萃分析的結果**

與伊托必利相比, 針灸亦可以更有效地改善功能性消化不良整體症狀 (合併RR: 1.30, 95%CI: 1.11 to 1.52) 。


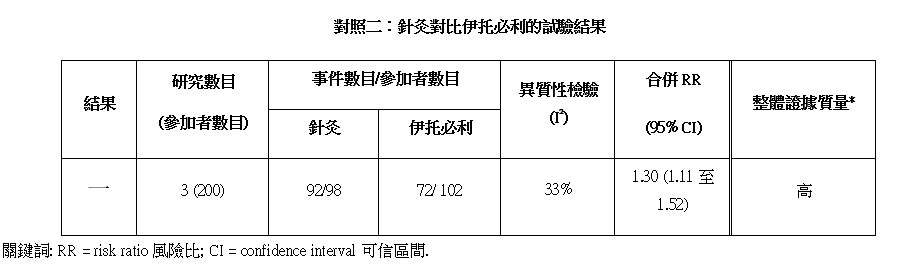


**對照三 不同針灸方案+胃腸動力藥對比只用胃腸動力藥**

**主要結果**

**網絡薈萃分析的結果**

有關治療後功能性消化不良整體症狀情況的改變，網絡薈萃分析發現11個不同針灸方案+胃腸動力藥的相對療效之間有統計學差異。

各種療法的相對療效排行如下:

| **排行** | **治療方案** | **最常用穴位** |
| --- | --- | --- |
| 一 | 針灸+氯波必利 | 足三里,內關,天樞 |
| 二 | 針灸+艾灸+多潘立酮 | 中脘,足三里,氣海,內關, 公孫 |
| 三 | 氯波必利 | 不適用 |
| 四 | 針灸 | 足三里,中脘,內關,太冲,內庭 |
| 五 | 艾灸 | 中脘,氣海,內關,公孫,胃俞 |
| 六 | 針灸+莫沙必利 | 足三里,內庭,太冲,內關,胃俞 |
| 七 | 電針灸 | 足三里,中脘,三陰交,合谷,內關 |
| 八 | 針灸+艾灸 | 中脘,內關,足三里,天樞,三陰交 |
| 九 | 多潘立酮 | 不適用 |
| 十 | 莫沙必利 | 不適用 |
| 十一 | 伊托必利 | 不適用 |

**結論**

**效果**

與單用多潘立酮或單用伊托必利相比, 各針灸療法可以更有效地改善功能性消化不良整體症狀。

相對來說, 針灸+氯波必利可能為最有效的治療方案。

**不良反應和副作用**

研究中有2個納入的試驗提及了不良反應和副作用, 它們都是輕微和暫時性的。

1個試驗報告針灸+艾灸治療後, 穴位處出現血腫, 而服用多潘立酮後出現皮疹及便秘症狀。另外1個試驗報告針灸+莫沙必利及只用莫沙必利治療後, 都分別出現輕微噁心、大便次數增加及腸鳴症狀。

**原文鏈結**

<https://www.ncbi.nlm.nih.gov/pubmed/28871092/>

**本概要源於以下文章:**

Ho RS, Chung VC, Wong CH, Wu JC, Wong SY, Wu IX. Acupuncture and related therapies used as add-on or alternative to prokinetics for functional dyspepsia: overview of systematic reviews and network meta-analysis. Scientific reports. 2017;7(1): 10320.

***整體證據質量5個等級的含義:**

• 極低: 進一步的研究非常可能改變目前效應估計值的可信性。

• 低: 進一步的研究很可能改變目前效應估計值的可信性。

• 中等: 進一步的研究可能改變目前效應估計值的可信性。

• 高: 進一步的研究不太可能改變目前效應估計值的可信性。

• 極高: 進一步的研究極少可能改變目前效應估計值的可信性。

有關證據質量評估的詳情可參考 Chung VC, Wu XY, Ziea ET, Ng BF, Wong SY, Wu JC. Assessing internal validity of clinical evidence on effectiveness of CHinese and integrative medicine: Proposed framework for a CHinese and Integrative Medicine Evidence RAting System (CHIMERAS). European Journal of Integrative Medicine. 2015 Aug 31;7(4):332-41.
