# Supplementary material for: Barriers and facilitators to promoting evidence uptake in Chinese medicine: a qualitative study in Hong Kong
Source: BMC Complement Med Ther. 2021 Jul 15;21:200. doi: 10.1186/s12906-021-03372-5 (PMC8280573; doi:10.1186/s12906-021-03372-5)
Supplement: Supplementary file 2 — Additional file 2. [file 12906_2021_3372_MOESM2_ESM.docx]

**Additional file 2. Semi-structured interview guide with Chinese medicine practitioners**

-------------------------------- **Intervention Characteristics**----------------------------------

- Evidence Strength & Quality

1. How do influential stakeholders (administrative or other leaders) think of the use of results from synopses in routine practice?

• What kind of supporting evidence or proof is needed about the effectiveness of synopsis to get staff on board?

2.What kind of evidence are you aware of that shows the use of results from synopses would, or would not work in your setting?

3.How will the positive experiences of applying results from synopses among medical doctors influence your decision to use them as well?

-Relative Advantage

4.How does the use of results from synopses in routine practice compare to other alternatives that may have been considered, e.g. applying knowledge learnt from the classical CM books, clinical experience or other sources of information in practice?

• What advantages and disadvantages does the use of results from synopses have?

- Adaptability

5.What kinds of changes or alterations will you make to the use of results from synopses so it will work effectively in your setting?

• Do you think you will be able to make these changes? Why or why not?

6. Who will decide (or what is the process for deciding) whether changes are needed to implement the use of results from synopses, so that it works well in your setting?

• How will you know if it is appropriate to make any changes?

Complexity

7.How complicated is the use of results from synopses in your routine practice? Please consider the following aspects of implementation the evidence-based CM intervention: duration, scope, intricacy and number of steps involved. Does the implementation of the use of results from synopses reflect a clear departure from previous practices?

Design Quality & Packaging

8.What is your perception of the quality of the existing available EBHC resources related to CM? Please use the four synopses as examples to elaborate.

• Why?

• Will you be able to access the full text of clinical evidence?

Cost

9. What costs will be incurred to implement the use of results from synopses?

• What cost were considered? e.g. opportunity costs, money costs, etc.

**-------------------------------Outer Settings--------------------------------------------**

Patient Needs & Resources

10. Do patients expect an EBHC approach when consulting a CMP?

11. What concerns do you think patients will have when you apply results from synopses in routine practice?

12. How essential is use of results from synopses to meet the patients’ needs?

Peer Pressure

13. Do you know any other Chinese medicine practitioners (CMPs) who have implemented EBHC approach in routine practice?

• What do they think? How did their thoughts influence your decision to use results from synopses?

14. To what extent would implementing the use of results from synopses provide an advantage for your organization, as compared to other providers?

• Is there a competitive advantage?

• Is there something about the use of results from synopses that would bring more patients into your organization?

External Policies & Incentives

15. Is there any local, state, or national performance measures, policies, regulations, or guidelines influenced the decision to implement the use of results from synopses?

16. What kind of financial or other incentives influenced the decision to implement the use of results from synopses?

• How will the use of the results from synopses affect revenue of your organization?

Cosmopolitan

17. To what extent do you network with other Chinese Medicine Practitioners outside your setting?

o What are the venues?

18. What kind of information exchange do you have with others outside your setting, either related to the use of evidence in routine practice, or more generally about your profession?

o What professional networking do you engage in? Listservs? Local or national conferences? Trainings?

19. To what extent does your organization encourage you to network with colleagues outside your own setting?

o Are you able to attend local/national conferences? Other venues?

**-----------------------------------------Inner Settings--------------------------------------------**

Networks & Communications

20. Can you describe your working relationship with i) leaders and ii) influential stakeholders on provision of evidence-based CM service?

Culture

21. How would you describe the culture of your organization or your own setting or unit, and how this affect the application of EBHC approach?

22. To what extent are new ideas being embraced, and be applied for making improvements in your organization?

• Can you describe a recent example regarding the application of EBHC approach?

23. How will your organization's culture (general beliefs, values, assumptions that people embrace) affect the implementation of the use of results from synopses?

• Can you describe an example which highlights this?

Implementation Climate

-Tension for Change

24. How essential is the use of results from synopses to meet the organizational goals and objectives?

-Compatibility

25. How well does the use of results from synopses fit with existing work processes in your setting?

• Which issues or complications may arise in the process?

-Relative Priority

26. To what extent might the use of results from synopses take a backseat to other high-priority initiatives going on now? Why?

-Organizational Incentives & Rewards

27. What kinds of incentives will help ensure that the implementation of the use of results from synopses is successful?

• What is your motivation?

Readiness for Implementation

-Leadership Engagement

28. What level of involvement has leadership at your organization had so far in your routine practice?

-Available Resources

29. Do you think that EBHC education workshop makes the practice of EBHC easier for you? Why?

30. We are going to develop an EBHC support service by publishing the critically appraised synopses in a searchable website. This may provide results of synopses to help answer your clinical queries.

• What do you think about this idea?

31. Do you have any other comments or suggestions regarding the application of EBHC approach in CM practice?

Structural Characteristics

32. How will the infrastructure of your organization (social architecture, age, maturity, size, or physical layout) affect your application of results from synopses?

o How will the infrastructure facilitate/hinder the use of results from synopses?

o How will you work around structural challenges?

Goals and Feedback

33. Have your organization set goals related to the application of results from synopses?

o [If yes] What are the goals?

Learning Climate

34. Can you describe a recent quality improvement initiative, or an implementation of a new program related to your routine practice?

35. Can you describe the new initiative/program and the motivation to improve/implement it?

o Can you tell me the major milestones or key accomplishments along the way?

o What factors helped make it successful/fail?

o Who were the key "players"?

o What was your involvement?

o Were people happy with the outcome/initiative?

o Can you tell me about how leaders were involved? Who? Their roles? How they helped/hindered?

Access to Knowledge & Information

36. Who do you ask if you have questions about use of the results from synopses?

o How available are these individuals?

**------------------------------Characteristics of Individuals ----------------------------------**

Knowledge & Beliefs about the Intervention

37. Did you learn evidence-based healthcare (EBHC) before?

• If yes, how do you define EBHC?

• Where did you learn? During your undergraduate study or continuing education?

• If no, do you think it is important to learn?

38. What do you think about the concept of evidence in Chinese medicine (CM)?

39. According to what you understand by EBHC, do you think there are any similarities/ differences between CM and EBHC? What are they?

40. How do you feel about the application of results from synopses of clinical evidence in routine practice in your setting?

• Do you think the application of results from synopses will be effective in your setting? Why?

• To what extent do you feel like you can try new things to improve your work processes?

• Do you have time and energy to think about ways to improve your work processes?

• How do you feel about the plan to apply results from synopses in routine practice in your setting?

• Do you have any feelings of anticipation? Stress? Enthusiasm? Why?

Self-efficacy

41. How much confidence do you think your colleagues will have on applying results from synopses? The scale ranges from 1 (very unconfident) to 5 (very confident), how will you rate?

• What gives them that level of confidence (or lack of confidence)?

Individual Stage of Change

42. Which stage of preparation are you at on applying results from synopses?

 Knowledge stage (Precontemplation) - knowledge of key aspects of the application of results from synopses

 Persuasion stage (Contemplation) - likes the application of results from synopses, discusses it with others, buys into it, has a positive view

 Decision stage (Preparation) - intends to seek additional information and try it

 Implementation stage (Action) - acquires additional information, uses results from synopses regularly, and has continued use

 Confirmation stage (Maintenance) - recognizes benefits, has integrated the application of results from synopses into routines, promotes use to others
